# Understanding of Abdominal Compartment Syndrome among Pediatric Healthcare Providers

**DOI:** 10.1155/2010/876013

**Published:** 2010-08-09

**Authors:** J. Chiaka Ejike, Jennifer Newcombe, Joanne Baerg, Khaled Bahjri, Mudit Mathur

**Affiliations:** ^1^Department of Pediatrics, Division of Pediatric Critical Care, School of Medicine, Loma Linda University, 11175 Campus Street, Suite A1117, Coleman Pavilion, Loma Linda, CA 92354, USA; ^2^Department of Nursing, Loma Linda University Children's Hospital, Loma Linda, CA 92354, USA; ^3^Department of Surgery, School of Medicine, Loma Linda University, Loma Linda, CA 92354, USA; ^4^Department of Epidemiology and Biostatistics, School of Public Health, Loma Linda University, Loma Linda, CA 92354, USA

## Abstract

*Background*. The sparse reporting of abdominal compartment syndrome (ACS) in the pediatric literature may reflect inadequate awareness and recognition among pediatric healthcare providers (HCP). *Purpose*. To assess awareness of ACS, knowledge of the definition and intraabdominal pressure (IAP) measurement techniques used among pediatric HCP. *Method*. A written survey distributed at two pediatric critical care conferences. *Results*. Forty-seven percent of 1107 questionnaires were completed. Participants included pediatric intensivists, pediatric nurses, and others. Seventy-seven percent (*n* = 513) of participants had heard of ACS. Only 46.8% defined ACS correctly. The threshold IAP value used to define ACS was variable among participants. About one-quarter of participants (83/343), had never measured IAP. *Conclusion*. Twenty-three percent of HCP surveyed were unaware of ACS. Criteria used to define ACS were variable. Focused education on recognition of ACS and measuring IAP should be promoted among pediatric HCP.

## 1. Introduction

The World Society of the Abdominal Compartment Syndrome (WSACS) has developed definitions for intraabdominal hypertension (IAH) and abdominal compartment syndrome (ACS) and outlined standardized techniques for intraabdominal pressure (IAP) monitoring to facilitate research and improve patient care [1]. ACS is defined as the presence of sustained IAP of 20 mmHg or greater (with or without an abdominal perfusion pressure of <60 mmHg) that is associated with new organ dysfunction or failure. IAH is defined as a sustained or repeated pathological elevation in IAP ≥ 12 mmHg [1, 2]. 

ACS has a clinically significant direct adverse effect on organ function and mortality despite its apparently low incidence of 0.9% to 12% [3–7]. It is an independent predictor of mortality. The mortality rate associated with ACS ranges from 50%–80% depending on the population studied and the definition of ACS used. It is also associated with a wide range of diseases seen in the ICU [3–5, 7]. 

**Figure 1:**
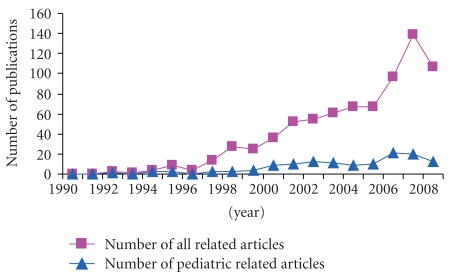
Trends of publications in Pubmed related to abdominal compartment syndrome (1990–2008).

It is important that pediatric healthcare providers (HCP) understand how to recognize, manage, and most importantly prevent ACS in those at risk to minimize the morbidity and mortality associated with it. Publications in children related to this field have greatly lagged behind those involving the adult population (Figure 1) reflecting less awareness, knowledge, or interest among pediatric HCP. 

The objective of this study was to assess the awareness of ACS as an entity, the understanding of the definition of ACS among pediatric HCP, and IAP measurement techniques currently being used.

## 2. Materials and Methods

The Institutional Review Board of Loma Linda University approved this study. A pilot study was first conducted to validate the questionnaire used. The questionnaire validity was checked by the correlation between items addressing the same objectives. In addition, factor analysis was used to assess the construct validity of the questionnaire. The internal consistency of ACS awareness achieved significance (*P* < .001) and Cronbach's alpha coefficient of 0.86. The results of Cronbach's alpha showed between 89.5% and 100% agreement on questions addressing similar items. For questions where minimum agreement was expected, the degree of agreement ranged from 9.1% to 16.7%. After validation of the questionnaire as an adequate tool designed specifically to assess pediatric HCP' awareness of ACS, the 10-item written questionnaire was administered at a national Pediatric Critical Care Nursing conference in 2006 and at the World Congress of Pediatric Critical Care in 2007. Voluntary completion of the survey was an indication of consent to study participation. It was distributed at the beginning of sessions not related to education on IAH, ACS, or related topics and collected at the end of each session. In fact, no topics related to IAH or ACS were part of the formal program at either of these conferences. The survey questions elicited information that relates to the awareness of ACS (one question), criteria for recognizing it, how IAP is currently being monitored, and experience in managing ACS among pediatric HCP (four questions). The years of ICU experience, type of practice such as tertiary or community hospital, and the place of practice were also elicited. Data derived from the questionnaires were entered into an excel spreadsheet for subsequent analysis.

## 3. Statistical Analysis

Descriptive statistics of the categorical variables were described as count and percent. Multivariable logistic regression was performed to assess the determinants of ACS awareness, measurement of IAP and knowledge of the ACS definition. Univariable logistic regression was used to identify the univariate effect of the potential determinants of the outcome. Significant variables were then put in a multivariable analysis to assess the significant variables after adjusting for all the other variables in the model. All statistical analyses were performed using SPSS statistical software version 17.0 (SPSS Institute Inc). Statistical significance was set at *P* of .05.

## 4. Results

Of 1107 questionnaires distributed, 517 (46.7%) were completed and returned. 

### 4.1. Description of the Respondents

Participants included General Pediatricians, Pediatric Registered Nurses (RN), Pediatric Intensivists (PCCM), and other providers (Pediatric Surgeons, Neonatologists, Pediatric Cardiologists, Pediatric Pulmonologists, Anesthesiologists, and Physician Assistants). More than half of the participants (57.1%) practiced in the USA/Canada compared to 26.1% in Europe and 16.8% in other places (Table 1). The other places included Asia 4.9%, Australasia 3.5%, South America 2.7%, Africa 1%, and the Middle East 2.7%. Eighty percent of responders practiced in a tertiary care hospital and 88.6% of the respondents worked in an ICU setting. Almost half (47.3%) of them had been in practice for more than ten years.

### 4.2. Awareness of ACS

Of all the HCP that participated in the study 77.8% (399/513) indicated that they had heard of ACS. The place, type and length of practice did not influence the awareness of ACS (Table 2). However, participants working in ICUs demonstrated a greater awareness of ACS. They were 2.9 (95% CI 1.60, 5.37) times more likely to have heard of ACS than those working outside of the ICU (Table 3). Sixty-six percent of HCP (264/399) who indicated awareness of ACS indicated personal experience in management of a child (<18 years) with ACS.

Pediatric intensivists demonstrated the greatest awareness of ACS among the professionals that participated in the survey. Ninety-seven percent of 153 pediatric intensivist respondents had heard of ACS. They showed more awareness than pediatric nurses and other specialties 95.0% and 89.9% of the time, respectively, as demonstrated by the odds ratios presented in Table 3.

### 4.3. Measurement of IAP and Experience in Management of Pediatric ACS Patients

Of the HCP that were aware of ACS, 24.2% (83/343) indicated that they had never measured IAP. Pediatric intensivists were more likely to measure IAP 44.9% of the time than nurses and 64.9% more often than other subspecialties (Table 3). The place, type, and length of practice did not influence likelihood of measuring IAP (Table 2). 

When IAP was measured, the method used most commonly was the intravesical technique, 210/311 (67.5%). Clinical exams alone were used by 20.3% (63/311) to detect IAP elevation. Other methods used less frequently included the direct method, the intraesophageal technique, and renal perfusion Doppler.

### 4.4. Knowledge of the Definition of ACS

The definition of ACS was characterized as an elevation of IAP by a specific number (threshold) in 53.2% (157/295) of respondents without indicating a need for evidence of new organ dysfunction as well. Only 46.8% understood that ACS is an elevation in IAP along with new multiorgan dysfunction. Pediatric intensivists understood the current definition of ACS more often than pediatric nurses by 55.8% but there was no significant statistical difference in understanding the definition when compared to other pediatric specialists (Table 3).

## 5. Discussion

This is the first survey targeting pediatric HCP directly and assessing their awareness and understanding of ACS. 

Our study demonstrates a lower awareness (77.8%) among pediatric HCP overall compared to adult ICU and surgical counterparts where awareness ranges of 80%–98.5% have been reported [8–10]. However, ACS awareness among pediatric intensivists (97.4%) was higher than other pediatric HCP and similar to the adult data. 

HCP working in ICUs are more likely to encounter patients at risk for developing ACS and showed more awareness of this entity than those that worked outside of the ICU. 

### 5.1. Knowledge of the Definition of ACS

Of all the questions in the survey, the question related to knowledge of the definition of ACS had the least participation (only 57% of participants). Most pediatric HCP in our survey incorrectly defined ACS as an elevation in IAP using a specific number alone. Only 46.8% understood that the development of new organ dysfunction/failure in addition to an elevation in IAP constituted the definition of ACS [4, 11–15]. Pediatric intensivists understood the definition of ACS more accurately compared to pediatric critical care nurses but no significant difference was seen when they were compared to the other subspecialists. Nonetheless overall knowledge of the definition was low. This is of concern as the interventions to treat ACS may not be appreciated by providers who do not recognize the syndrome or do not have a good understanding of its pathophysiology. 

It is important to understand that ACS is the end of the spectrum of IAH [16]. Different grades of IAH exist (grade I: IAP 12–15 mmHg, grade II: IAP 16–20 mmHg, grade III: IAP 21–25 mmHg, grade IV: IAP > 25 mmHg) [1, 2] but ACS is an all or nothing phenomenon that is present when there is elevation in IAP along with new organ dysfunction irrespective of the actual IAP number. 

In fact, prior to the emergence of the consensus definitions, there was great variability in thresholds for defining ACS, ranging from 11–40 mmHg within and between different specialties [6, 8, 10, 12, 13, 17–20]. Our study found that confusion persists amongst pediatric HCP regarding the definition of ACS. The lower response rates to the question related to knowledge of the definition may indicate uncertainty among the nonresponders about the definition of ACS. Some of this confusion may stem from the variety of patient ages and sizes that pediatric HCPs encounter on a daily basis. Kimball's study had reported that 38% of pediatric intensivists believed that the threshold IAP to cause physiologic compromise was patient dependent compared to 7%–17% of all other specialties surveyed [18]. This belief is understandable considering the pediatric context. Patients range from newborn babies that weigh approximately 3.5 kg to adult-sized teenagers weighing 70 kg or more with an equally wide range of blood pressures. Elevated IAP mechanically affects perfusion of blood to intra- and extraabdominal organs, and any evidence of new organ dysfunction is the important component of the clinical assessment that transforms IAH into ACS. Abdominal perfusion pressure is defined as mean arterial pressure (MAP) minus IAP [1, 2, 21]. Therefore organ dysfunction occurring as a result of decreased organ perfusion may occur more frequently at IAP less than 20 mmHg in children by virtue of their MAP generally being lower than adult's. The critical threshold for APP currently associated with the ACS definition is 60 mmHg for adults [1, 2, 21]. Although this is not an absolute requirement to meet the definition of ACS, one can see how this definition cannot be directly applied to the pediatric patient whose MAP even under normal conditions may not reach 60 mmHg. Pediatric HCP may then understandably be confused by a generalized IAP threshold as part of the ACS definition. 

To our knowledge, there are no studies examining the APP threshold in children associated with organ dysfunction or ACS. Critical APP associated with new organ dysfunction may be of greater significance for defining ACS in the pediatric population than an actual IAP value due to the wide range of MAPs seen in children. It may be more practical to define ACS as a rising or sustained elevation of IAP above normal along with the development of new organ dysfunction [5, 14, 16, 22]. Results from our survey highlight not only the need for better education of pediatric HCPs but also perhaps the need for establishing clearer definitions of IAH and ACS specific to children.

### 5.2. Measurement of IAP

Measuring and monitoring IAP is fundamental to recognizing, diagnosing, and managing IAH/ACS appropriately. IAH occurs more frequently than ACS and has been identified as an independent predictor of morbidity and mortality among the critically ill [4, 7, 23, 24]. Therefore HCP must be knowledgeable on how to measure and monitor IAP. 

Early intervention should be directed at lowering elevated IAP before organ damage occurs [25]. Our study showed that 24.2% of HCP aware of ACS had never measured IAP. In previous studies, there was a wide variability (ranging from 6%–89%) in the percentage of HCP who routinely or frequently measure IAP [8–10, 12, 13, 18, 19, 26]. A moderate number of HCP still believe that they can diagnose IAH or ACS by physical examination alone. Our study showed that 21.9% of pediatric HCP indicated that they monitored IAP by clinical exam alone. Tiwari et al. found that 60% of intensivists in tertiary settings and 76% in general hospitals measured IAP solely by clinical exam. Studies have shown that a clinical estimation of IAP by abdominal girth or by examiner's feel of the tenseness of the abdomen is far from accurate, with a sensitivity of around 40% [27–30]. Our findings suggest that HCP need education on objective IAP measurement techniques essential for the detection of IAH or ACS. 

Among the study participants that did measure IAP, the intravesical method was most often used. This was also the preferred technique described in other surveys done amongst adult HCP [8–10, 12, 13, 18, 19, 26]. 

Of the participants that were aware of ACS, 33.3% (132/396) had never managed a child with ACS. A survey amongst trauma surgeons found that busier trauma surgeons, regardless of age or academic appointment, and those who measure IAP were more likely to have recent experience with ACS. An overwhelming majority of those who indicated they never or rarely measure IAP stated they had not diagnosed ACS in the previous year [13]. These findings suggest that surveillance for IAH and ACS by IAP monitoring increases detection. Early and appropriate intervention may result in more focused management and in turn prevent or reduce the morbidity and mortality known to be associated with ACS.

### 5.3. Limitations to the Study

The low response rate in our survey is a limitation and may suggest that the findings may not be accurately reflective of the study population. Persons directly interested in the subject or those with strong opinions regarding the subject may have responded, introducing a selection bias. Other eligible participants not knowledgeable or not interested in the subject might not have participated at all. Some others who participated provided incomplete responses, reducing the strength of our conclusions. For instance, there was a 100% response rate to questions that asked about personal factual information such as profession, place of practice, and type of practice and even regarding whether or not they had heard about ACS or had managed a child with ACS. However the questions addressing personal clinical practice patterns or specific knowledge regarding the subject generated a response rate that varied from 74 to 86%. These questions might have been perceived as having a “right or wrong answer” making the respondents who were not sure of the “expected” answer unwilling to respond. The reduced response rates to certain questions may be interpreted as a lack of knowledge or comfort with the subject or “survey fatigue” even though the survey consisted of only 10 questions. Another limitation was that the survey was conducted soon after the emergence of the consensus definitions, probably before the new definitions could be adequately disseminated. Nonetheless our study highlights that further education regarding ACS is necessary to improve the existing knowledge among pediatric HCP.

## 6. Conclusion

The majority of pediatric HCP surveyed were aware of ACS, with pediatric intensivists having the greatest awareness. Definitions of ACS specific to children are needed. Further dissemination of knowledge related to the importance of objective monitoring of IAP is necessary for diagnosis and early recognition of ACS among pediatric HCP.

## Figures and Tables

**Table 1:** Descriptive statistics of responder demographics.

Profession	*n* = 513	Percentage
General Pediatrician	20	3.9
Pediatric Registered Nurse	307	59.8
Pediatric Intensivist	155	30.2
Others	31	6.1

Type of Practice	*n* = 504	Percentage
Tertiary/Teaching Institution	407	80.8
Community Hospital	72	14.3
Private Practice	10	2.0
Clinics	4	0.8
Others	11	2.2

Place of Practice	*n* = 513	Percentage
USA/Canada	293	57.1
Europe	134	26.1
Others	86	16.8

Years in Practice	*n* = 512	Percentage
0 to 5 years	149	29.1
>5 to 10 years	121	23.6
Greater than 10 years	242	47.3

**Table 2:** Univariable analysis of factors affecting ACS awareness, IAP measurement, and knowledge of ACS definition.

	ACS Awareness	IAP Measurement	ACS Definition
Determinants	OR (95% CI)	OR (95% CI)	OR (95% CI)
Institution Type			
Tertiary versus others	1.196(0.463,3.088)	2.344(0.844,6.510)	1.709(0.488,5.983)
Community versus others	0.821(0.287,2.353)	0.926(0.295,2.911)	0.737(0.168,3.238)

ICU			
Yes versus No	3.370(1.911,5.944)*	2.365(1.110,5.040)*	1.223(0.477,3.135)

Profession			
Nurses versus PCCM	0.057(0.021,0.160)*	0.548(0.330,0.910)*	0.480(0.293,0.788)*
Others versus PCCM	0.082(0.025,0.265)*	0.335(0.158,0.708)*	1.369(0.618,3.037)

Length of Practice			
6–10 yrs versus <5 yrs	1.618(0.906,2.887)	1.481(0.784,2.800)	1.530(0.799,2.931)
>10 yrs versus <5 yrs	1.458(0.906,2.345)	0.672(0.394,1.146)	0.891(0.513,1.547)

Place of Practice			
Europe vesus USA	1.912(1.132,3.230)*	1.526(0.882,2.642)	1.224(0.726,2.065)
Others vesus USA	2.316(1.195,4.490)*	1.196(0.669,2.141)	2.031(1.099,3.756)*

*Significant at an alpha of 0.05.

ACS: Abdominal compartment syndrome, IAP: Intraabdominal pressure, OR: Odds ration, CI: Confidence interval, ICU: Intensive care unit, PCCM: Pediatric Intensivist.

**Table 3:** Multivariable analysis of factors affecting ACS awareness, IAP measurement, and knowledge of ACS definition.

	ACS Awareness	IAP Measurement	ACS Definition
Determinants	OR (95% CI)	OR (95% CI)	OR (95% CI)
ICU			
Yes versus No	2.936(1.604,5.372)*	2.085(0.965,4.507)	—

Profession			
Nurses versus PCCM	0.050(0.017,0.149)*	0.551(0.330,0.922)*	0.442(0.241,0.811)*
Others versus PCCM	0.101(0.031,0.332)*	0.351(0.164,0.750)*	1.534(0.681,3.458)

Place of Practice			
Europe versus USA	0.656(0.334,1.288)	—	0.689(0.359,1.323)
Others versus USA	0.790(0.359,1.738)	—	1.258(0.621,2.549)

*Significant at an alpha of 0.05.

ACS: Abdominal compartment syndrome, IAP: Intraabdominal pressure, OR: Odds ratio, CI: Confidence interval, ICU: Intensive care unit, PCCM: Pediatric Intensivist.
